# Green Electrospinning of Polymer Latexes: A Systematic Study of the Effect of Latex Properties on Fiber Morphology

**DOI:** 10.3390/nano11030706

**Published:** 2021-03-11

**Authors:** Edurne Gonzalez, Aitor Barquero, Belén Muñoz-Sanchez, María Paulis, Jose Ramon Leiza

**Affiliations:** POLYMAT, Kimika Aplikatua Saila, Kimika Fakultatea, University of the Basque Country UPV/EHU, Joxe Mari Korta Zentroa, Tolosa Hiribidea 72, 20018 Donostia-San Sebastián, Spain; aitor.barquero@ehu.eus (A.B.); belen.munozsa@gmail.com (B.M.-S.); maria.paulis@ehu.eus (M.P.); jrleiza@ehu.eus (J.R.L.)

**Keywords:** electrospinning, latex, green electrospinning, nanofibers, fiber morphology

## Abstract

Green electrospinning is a relatively new promising technology in which a polymer (latex) can be spun from an aqueous dispersion with the help of a template polymer. This method is a green, clean and safe technology that is able to spin hydrophobic polymers using water as an electrospinning medium. In this article, a systematic study that investigates the influence of the template polymer molar mass, the total solids content of the initial dispersion and the particle/template ratio is presented. Furthermore, the influence of the surfactant used to stabilize the polymer particles, the surface functionality of the polymer particles and the use of a bimodal particle size distribution on the final fiber morphology is studied for the first time. In green electrospinning, the viscosity of the initial complex blend depends on the amount and molar mass of the template polymer but also on the total solids content of the dispersion to be spun. Thus, both parameters must be carefully taken into account in order to fine-tune the final fiber morphology. Additionally, the particle packing and the surface chemistry of the polymer particles also play an important role in the obtained nanofibers quality.

## 1. Introduction

Electrospinning is a well-stablished technology used to create polymer nanofibers. This technology has gained extraordinary relevance in recent years due to its simplicity and low cost as well as the possibility to effectively scale it up opening perspectives for industrial production [1,2,3,4]. Electrospun nanofibers have exceptional properties such as a huge area to volume ratio, porous structure and tunable functionality. These unique properties make electrospun materials very attractive for a broad range of applications such as textiles, filters, tissue engineering, drug delivery, wound healing, sensors, environmental remediation, aerogels, dye adsorption, packaging, energy storage and catalysis, among others [5,6,7,8,9,10,11,12].

Solution electrospinning is the most widely used electrospinning method; however, it presents some limitations for its industrial application. The first limitation is the need to use toxic and flammable organic solvents, which can be problematic for industrial production due to more and more stringent environmental and safety regulations. As an alternative, water can be used as a solvent but in this way only water-soluble polymers can be electrospun, and therefore, the produced nanofiber material will also be water soluble, a fact that might be problematic for several applications. There are different crosslinking methods to increase the water resistance of water-soluble nanofibers, but they usually require high temperatures or toxic crosslinkers [13,14,15,16]. The second limitation is related to the polymer concentration of the electrospinning solution. There is a maximum critical concentration that can be used in this process, which is around 10–15 wt.% of polymer (depending on its molar mass). Polymer solutions of higher concentrations are not spinnable due to their high viscosity. This concentration limitation decreases the productivity of the electrospinning process significantly.

Suspension electrospinning, also named green electrospinning, is a novel and promising method that consists in the use of an aqueous polymer dispersion (latex) as an electrospinning solution with the help of a polymer template. This method overcomes the abovementioned limitations, as it allows the use of water as electrospinning medium, even for hydrophobic polymers and enables dispersion of higher polymer concentrations to be spun, increasing the overall productivity of the process [17,18]. 

The first paper on green electrospinning was published by Greiner and coworkers in 2007 [19]. They electrospun poly(styrene) (PS) polymer particles of different diameters using polyvinyl alcohol (PVA) as polymer template with the help of a polymeric surfactant (Basenol). Since then, different works have been published on the electrospinning of diverse types of polymer particles such as PS [20,21,22,23,24], poly(styrene-co-butyl acrylate) (PS/BA) [25], poly(methyl methcrylate-co-butyl acrylate) (PMMA/BA) particles bearing crosslinkable monomers [26,27], crosslinked core-shell PS/PMMA particles bearing quantum dots [28], waterborne polyurethanes (WPU) [29,30,31], microgels [32], core-shell microcapsules [33,34,35] or block copolymers [36]. Regarding the type of polymer template, although PVA has been the most frequently used one [19,20,21,22,23,24,25,26,27,28,29,30,31,37], others such as polyethylene oxide (PEO) [34,36] (or poly(vinyl formamide) (PVFA)) [26] have also been employed. These novel composite nanofibers obtained by green electrospinning have been claimed to have potential applications in tissue engineering, medicine, pharmacy, agriculture or sensor technology [28,30,32,33,36].

Although different particle/template systems have been successfully spun, there are very few works in the literature that thoroughly study the influence of the initial dispersion composition and properties on the final fiber morphology. Fiber morphology and water resistance have been demonstrated to be affected by the particle/template weight ratio [19,22,23,29], the particle diameter [19,22,25] and the glass transition temperature (Tg) [25] or the crosslinking of the polymer particles [26,27]. However, in order to be able to fine-tune the fiber morphology, the influence of the initial dispersion composition on the final fiber morphology must be well understood. To this end, a systematic study that investigates the effect of the template polymer molar mass, the total solids content of the dispersion (that is, the total concentration of polymer, template plus polymer particles) and the particle/template ratio in a single work is necessary and lacking in the literature. In this article, we present this systematic study. Furthermore, we have also investigated for the first time, the influence of another three parameters: the surfactant used to stabilize the polymer particles, the surface functionality of the polymer particles and the use of a bimodal particle size distribution.

The polymer particles used in this work were composed of a copolymer of MMA/BA in a 50/50 wt.%/wt.%. These particles are film forming (their Tg is below room temperature) [38], and therefore, they might coalesce within the fiber, forming a continuous phase [25]. The chosen template polymer was PVA. This work presents a systematic and detailed study on the influence of the initial electrospinning dispersion composition on the final nanofibers morphology. Electrospinning dispersions have been properly characterized by means of viscosity, surface tension and conductivity measurements. The morphology of the electrospun nanofibers have been assessed by Scanning Electron Microscopy (SEM).

## 2. Materials and Methods

### 2.1. Materials

**Figure 1:**
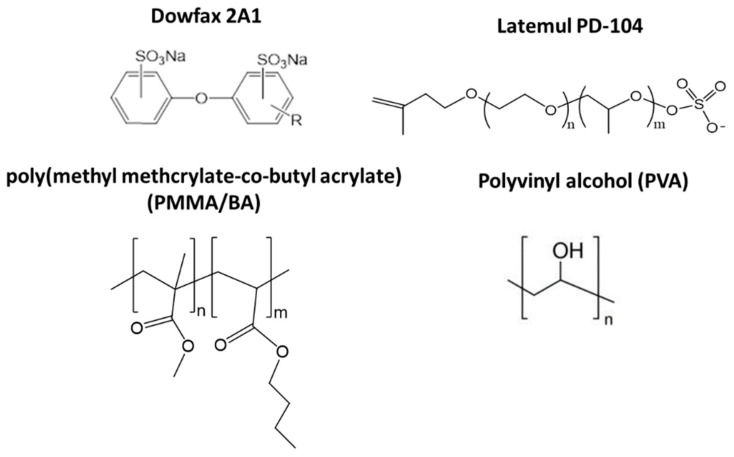
Chemical structure of the PVA template polymer, the latex copolymer (PMMA/BA) and the used surfactants to stabilize the polymer particles (Dowfax 2A1 and Latemul PD-104).

Three different polyvinyl alcohol (PVA) polymers were purchased from Kuraray to be used as template polymer (Table 1). Table 2 summarizes the properties of the different latexes used in this work; their synthesis procedure is explained in the Supporting Information. The chemical structures of the PVA template polymer, the latex copolymer and the used surfactants to stabilize the polymer particles are shown in Figure 1.

### 2.2. Preparation of Electrospinning Dispersions and Electrospinning Process

The electrospinning dispersions were prepared in 5 mL vials under magnetic stirring, adding the latex (with 50 wt.% s.c.) to the PVA aqueous solution (in a concentration of 10 wt.%) dropwise. In order to adjust the final s.c. of all the blends to 17 wt.%, in some cases water was added to the PVA solution before the latex was added (Table 3).

For the electrospinning experiments, polymer dispersions were placed into a syringe with an 18-gauge blunt-end needle that was mounted in a syringe pump (Cole-Parmer, Vernon Hills, IL, USA). Randomly oriented nanofibers were electrospun by applying a voltage of 15 kV to the needle using a Spellman CZE1000R high voltage supply (0–30 kV CZE1000R; Spellman High Voltage Electronics Corp. (Hauppauge, NY, USA)), with a low current output (limited to a few A). The ground plate (stainless steel) was placed at 15 cm from the needle tip. The syringe pump delivered the polymer solutions at a controlled flow rate of 1 mL/h. The temperature and relative humidity (R.H.) of the electrospinning chamber varied from 20 to 25 °C and from 31 ± 1% to 55 ± 1%, respectively. The exact temperature and R.H. is specified for each experiment.

### 2.3. Characterization Methods

Polymer particle average diameter (d_p_) was measured by dynamic light scattering (Zetasizer Nano Z, Malvern Instruments, Malvern, UK). The viscosity of all the blends was measured from 10 to 1000 s^−1^ in an Antor Paar rheometer using concentric cylinders. The surface tension of the blends was measured by means of the Du Noüy ring method using a KSV Sigma 700 tensiometer (Iberlaser, Madrid, Spain). Nanofiber morphology was analyzed by scanning electron microscopy (SEM) in a Hitach TM3030 Scanning Electron Microscope (Monocomp, Madrid, Spain). ImageJ™ open source software (National Institutes of Health, Bethesda, MD, USA) was used on the SEM images to measure the mean average fiber diameters. Fifty measurements were taken for each sample from three separate images.

## 3. Results and Discussion

### 3.1. Effect of the Template Polymer and the Solids Content (S.C.)

First, the influence of the PVA molar mass and amount on the final fiber morphology was studied. To this end, latex D_2 was blended with PVA polymers of different molar masses. The PVA/particle ratio was varied from 50/50 to 17/83 wt.%/wt.%. It is important to remark that the total solids content (s.c.) of all the dispersion, that is, the concertation of the total polymer (PVA plus polymer particles), was kept constant to 17 wt.% in all the cases. 

**Figure 2:**
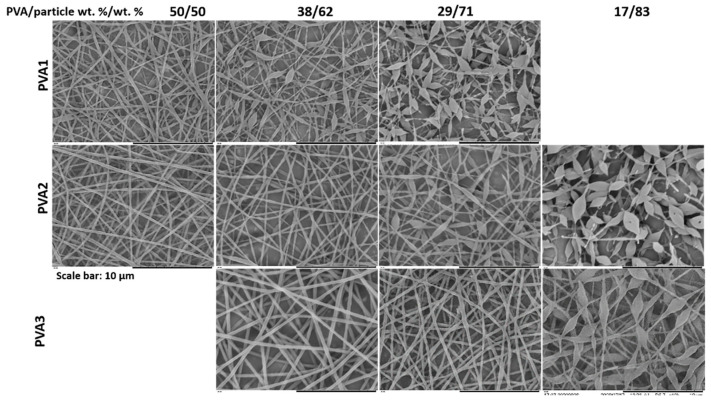
SEM images of fibers obtained from blends of latex D_2 and different concentrations of PVA with different molar masses. Total s.c. of the solutions was 17 wt.%. The experiment was performed at 22 °C and 31 ± 1% of R.H.

As it can be observed in Figure 2, at high PVA/particle ratios, uniform and continuous fibers were obtained. However, as the PVA amount was decreased, non-uniformities or beads started to appear in the fibers. The same trend was observed when PVA polymers of different molar masses were used as template (beads started to show at low PVA amounts). Interestingly, as the PVA molar mass increased, defects appeared at lower PVA concentrations. Therefore, controlling the increase in the molar mass of template polymer, it is possible to obtain bead-free fibers with lower template concentration.

Bead formation is usually considered as a defect and, therefore, is normally an undesired phenomenon. It is caused by the jet instability during the electrospinning process [39], and it is affected by the electrospinning solution properties (viscosity, surface tension and conductivity) as well as by the process parameters (applied voltage, tip to collector distance and flow rate) [1,4,40,41]. 

In this case, the process parameters used in all the experiments were exactly the same (see Materials and Method section); thus, the difference in the fiber morphology can only be due to the properties of the electrospinning dispersions. In conventional solution electrospinning, it is well known that bead formation tends to decrease by increasing the viscosity and/or conductivity of the solution as well as by decreasing its surface tension [1,41]. Table 4 shows the conductivity and surface tension of the blends prepared using PVA2 as template polymer. The surface tension values of all these blends were very similar, but the conductivity values slightly increased as the PVA amount was increased. In order to better understand these results, the conductivity of a PVA2 water solution and the one of latex D_2, both at 10 wt.% s.c., were also measured obtaining values of 758 and 364 µS/cm, respectively. Thus, when measuring the conductivity of D_2/PVA blends, the contribution of the amount of PVA is greater than the one of D_2. This explains why the conductivity of the blends increased as the PVA amount was increased. Phachamud et al. [42] investigated the effect of different process parameters in conventional PVA solution electrospinning and also observed that the conductivity of PVA water solutions increased with the PVA concentration.

**Figure 3:**
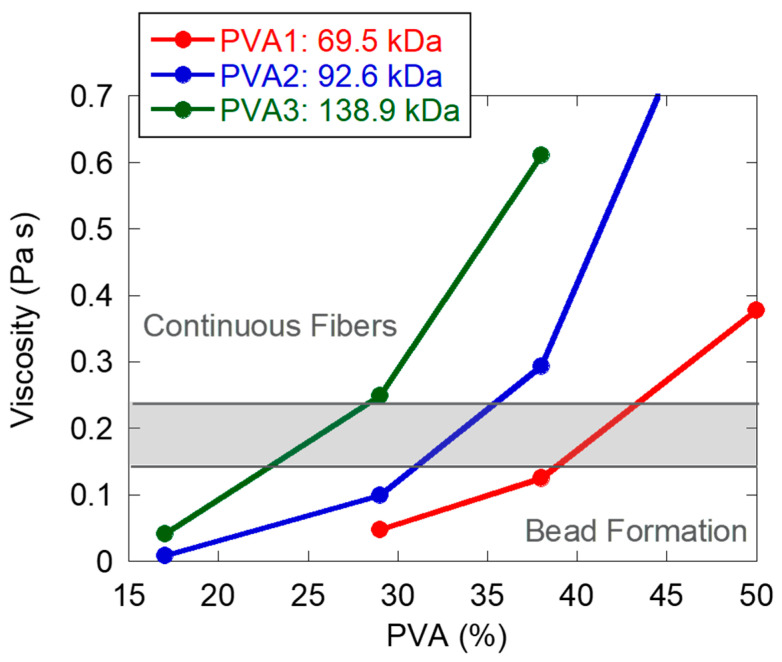
Viscosity of the electrospinning solutions (measured at 200 s^−1^) obtained blending latex D_2 with different concentrations of PVA with different molar masses. Total s.c. of the solutions was 17 wt.%.

The viscosity of all the blends shown in Figure 2 was measured from 10 to 1000 s^−1^ using concentric cylinders. In order to have an accurate comparison between the different samples, viscosity values should be compared at the shear rate at which the fiber formation during electrospinning process occurs. Since this shear rate value is unknown, values obtained at 200 s^−1^ were compared (Figure 3) in order to have a simple and systematic comparison. The viscosity values of the samples along all the shear rate range are shown in the Supporting Information. Figure 3 shows that, as expected, the viscosity of the solution increased as the molar mass and amount of PVA increased. Combining Figure 2 and Figure 3, a window of viscosity values (marked in grey in Figure 3) that separates beaded fiber morphology from uniform morphology can be drawn. According to this window, the higher the molar mass of the template, the lower the concentration of template required to produce uniform fibers. The minimum viscosity required to obtain continuous fibers was in the range of 0.14 and 0.23 Pa·s. Note that this viscosity window has been obtained with a limited number of solutions and, hence, is only qualitative.

These results are in agreement with the ones obtained by Yuan et al. [22] and Cao et al. [23], where PS nanoparticles were electrospun using PVA as polymer template. A beaded morphology was achieved at low PVA concentrations in both works, and it was attributed to the low viscosity of the electrospinning dispersions. However, only Yuan et al. [22] reported viscosity values, even if they did not specify the shear rate. Furthermore, none of these works studied the effect of the PVA molar mass.

Figure 2 also shows that for a given PVA type, bead size increased as the PVA concentration was decreased, that is, as the viscosity of the solution was decreased. Yuan et al. [22] also observed an increase in the bead size when the concentration of template polymer was reduced, and they attributed this phenomenon to a decrease in the solution viscosity.

**Figure 4:**
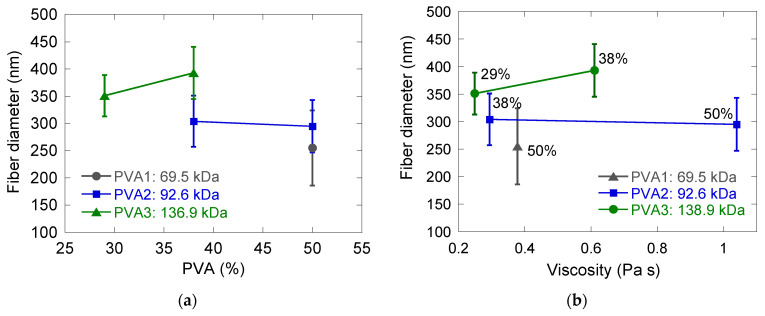
(**a**) Average fiber diameters as a function of the PVA amount; (**b**) average fiber diameter as a function of the viscosity of the electrospinning solution. Error bars correspond to the standard deviation. The number at each point indicates the PVA amount of each sample. In both cases, electrospinning solutions were blends of latex D_2 and different concentrations of PVA polymers of different molar masses. Total s.c. of all the solutions was 17 wt.%.

If only uniform, bead-free fibers are considered (Figure 2), it can be observed that the diameter of the fibers increased as the molar mass of the PVA template polymer was increased (Figure 4a). When PVA3 was used as polymer template, the average fiber diameter also increased when the amount of PVA was increased. In contrast, it was not possible to observe any clear trend when PVA2 was used as template polymer. Yuan et al. [22] and Wu et al. [30] reported an increase in final fiber diameter as the template/particle ratio was increased. Yuan et al. [22] attributed it to an increase in the electrospinning dispersion viscosity, while Wu et al. [30] just mentioned that it was caused by the different amount of template polymer in the aqueous phase.

One might think that the increase in the fiber diameter when the PVA of higher molar masses used could be due to an increase in the viscosity of the dispersion; however, this was not the case. No clear trend was observed when plotting the average fiber diameter as a function of the viscosity of the electrospinning dispersion (Figure 4b).

**Figure 5:**
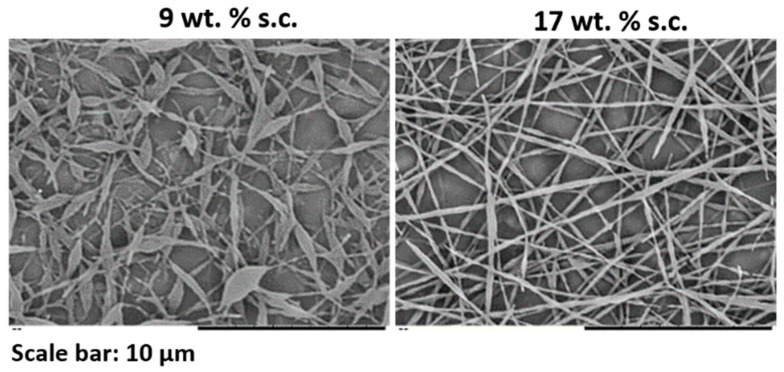
SEM images of fibers obtained from blends of latex D_2 and PVA2 (38 wt.%) at different total s.c. The experiment was performed at 20 °C and 55 ± 1% of R.H.

In a second step, blends of latex D_2 with the same PVA/particle ratio (38/62 wt.%/wt.%) were compared but containing different total s.c. Figure 5 shows the presence of beads for the solution at 9 wt.% s.c. and bead-free fibers when the s.c. was increased to 17 wt.%. Again, this was related to the viscosity of the electrospinning solutions. The viscosity of the solution at 9 wt.% s.c. (0.05 Pa·s) was below the critical viscosity window (between 0.14 and 0.23 Pa·s) defined above; hence, beaded fibers were produced. In contrast, the viscosity of the dispersion at 17 wt.% s.c. (0.29 Pa·s) was well above the critical viscosity, leading to uniform fibers.

It is well known that the viscosity of polymer solutions increases with polymer concentration and molar mass [43]. Thus, in conventional solution electrospinning, for a given polymer/solvent solution, bead formation can be avoided by just increasing the polymer molar mass or its concentration [1,41]. In green electrospinning, however, the electrospinning medium is not a simple polymer solution but a blend of a polymer dispersion and a water soluble polymer. Therefore, the viscosity of these complex dispersions is not only related to the total concentration of polymer (total s.c.), but also to the viscosity of the continuous phase (the template polymer in water) and to the particle size distribution and the particle concentration [44,45,46]. Thus, it can be concluded that in green electrospinning, these parameters must be carefully controlled in order to control the viscosity of the complex dispersion and consequently fine-tune the final fiber morphology.

### 3.2. Effect of Particle Size and Particle Size Distribution

**Figure 6:**
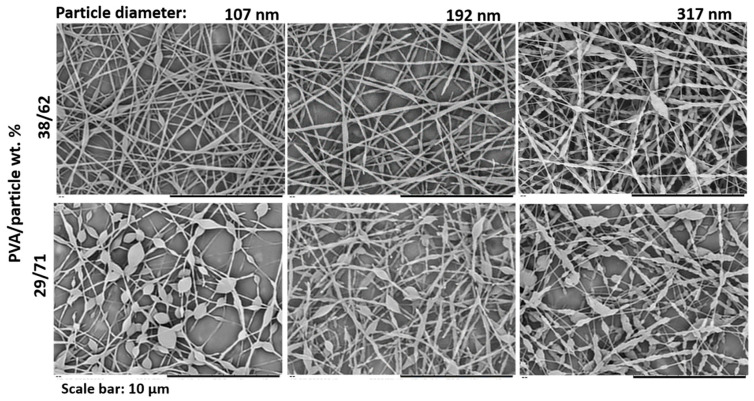
SEM images of fibers obtained from blends of latexes D_1 (107 nm), D_2 (192 nm) and D_3 (317 nm) with PVA2. The total s.c. was 17 wt.% in all the cases. The experiment was performed at 20 °C and 55 ± 1% of R.H.

In order to study the effect of the latex particle size on the final fiber morphology, latexes D_1 (107 nm), D_2 (192 nm) and D_3 (317 nm) were blended with PVA2. Dispersions with two PVA/particle ratio were prepared, 29/71 and 38/62 wt.%/wt.%. The total s.c. was 17 wt.% in all the cases. As it can be observed in Figure 6, when a PVA/particle ratio of 38/62 wt.%/wt.% was used, uniform fibers were obtained for the samples containing the latexes D_1 (107 nm) and D_2 (192 nm) but not for the samples containing the sample D_3 (317 nm) that showed a pearl necklace morphology. Since the viscosity of all the initial complex dispersions was very similar, around 0.3 Pa·s (all values are shown in the Supporting Information), the pearl necklace morphology was attributed to the worse packing of the bigger polymer particles. Although the particle packing along the fiber can be altered by the used template/particle ratio [22], it has been demonstrated that small polymer particles lead to a close packing arrangement along the fiber, whereas bigger particles lead to a one by one particle packing [19].

The average fiber diameter was also measured for fibers containing D_1 (107 nm) and D_2 (192 nm) particles, and values of 177 ± 26 and 264 ± 52 nm were obtained, respectively. Since the viscosity of both complex dispersions was very similar (around 0.3 Pa·s, see Supporting Information), it can be concluded that the larger the initial polymer particles, the larger the final fiber diameter. Greiner and co-workers [25] electrospun two PS/BA latexes of different particle size (153 and 102 nm) using PVA as polymer template. They also observed that larger polymer particles led to fibers with larger average diameter, but they did not report any viscosity values. 

**Figure 7:**
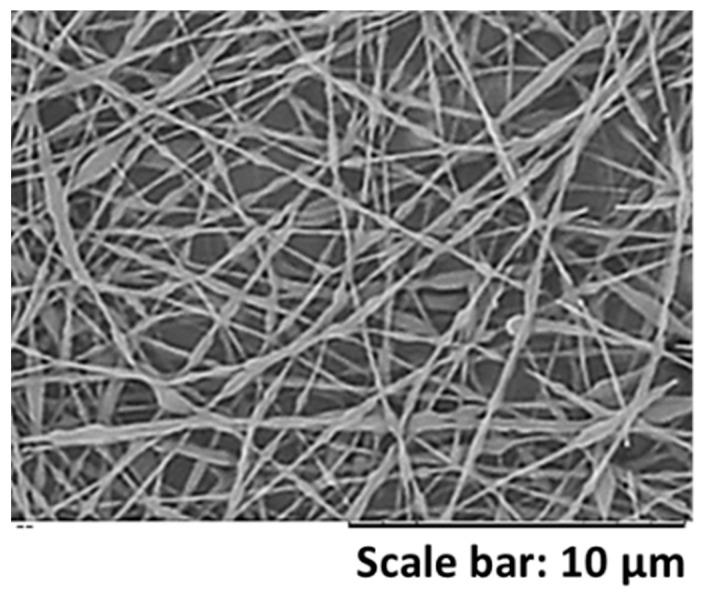
SEM images of fibers obtained from a mixture of latexes D_1 and D_3 in a 50/50 wt.%/wt.% ratio and blended with PVA in a PVA/particle ratio of 29/71 wt.%/wt.%. The total s.c. was 17 wt.%. The experiment was performed at 20 °C and 55 ± 1% of R.H.

When a PVA/particle ratio of 29/71 wt.%/wt.% was used, non-uniform beaded fibers were obtained with the three different particle sizes. With the objective to study, the effect that a bimodal particle size distribution could have on the fiber morphology, latexes D_1 and D_3 were mixed in a 50/50 wt.%/wt.% ratio and blended with PVA2 in a PVA/particle ratio of 29/71 wt.%/wt.%. Obtained fibers are shown in Figure 7. As can be observed, while a beaded morphology was obtained with the three monomodal latexes, significantly more uniform fibers were obtained when the bimodal latex was used. The viscosity values for the solutions with latexes D_1 and D_3 were 0.19 and 0.12 Pa·s, respectively, whereas the one for the bimodal latex was 0.35 Pa·s (values are shown in the Supporting Information). Therefore, the reason behind a more uniform fiber morphology was attributed to an increase in the blend viscosity. This is the first time that the effect of the use of a bimodal particle size system is investigated.

### 3.3. Electrospinning of Polymer Particles with Different Surface Chemistry

**Figure 8:**
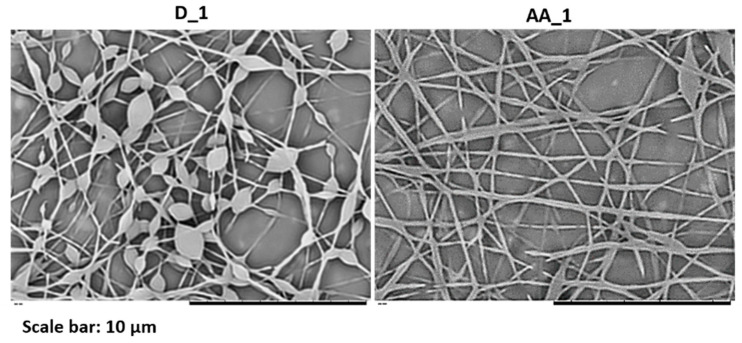
SEM images of fibers obtained from blends of latexes D_1 and AA_1 with PVA2. The amount of PVA was 29 wt.% and the total s.c. 17 wt.% in both cases. The experiment was performed at 20 °C and 55 ± 1% of R.H.

With the objective to analyze if the surface chemistry of the polymer particles have any influence on the final fiber morphology, polymer particles containing carboxylic acid groups in their surface and particles stabilized by different surfactants were electrospun. First, blends of latexes D_1 and AA_1 were electrospun and compared using the same conditions and process parameters (Figure 8). Both latexes were synthesized using the same surfactant and had similar average particle size; however, latex AA_1 contained 1 wbm % of AA in the latex formulation; thus, the polymer particles of this latex contained carboxylic acid groups on their surface [47]. The electrospinning dispersions were prepared blending both latexes with PVA2 in a PVA/particle ratio of 29/71 wt.%/wt.%. The solids content was 17 wt.% in both cases. 

Figure 8 shows that more uniform fibers were obtained when latex AA_1 was used (containing carboxylic acid groups at the particle surfaces) instead of D_1. Although both complex electrospinning dispersions showed a similar surface tension value, the dispersion containing latex AA_1 had higher conductivity and viscosity (Table 5). As it has been explained before, an increase in both parameters helps in reducing bead formation and, therefore, in obtaining more uniform fibers. In fact, whereas the viscosity of the blend containing D_1 latex is in the lower part of the critical viscosity window observed in Figure 3 (between 0.14 and 0.23 Pa·s), the one of the blend containing latex AA_1 is above it. Furthermore, it should be noted that film formation of latexes is influenced by the surface chemistry of the polymer particles [48,49,50,51]. Some authors have demonstrated that the presence of carboxylic acid groups on the particle surface can lead to a hydroplasticization phenomenon enhancing the film formation [52,53,54,55]. Thus, the better quality of the fibers containing latex AA_1 may also be due to this hydroplasticization effect.

**Figure 9:**
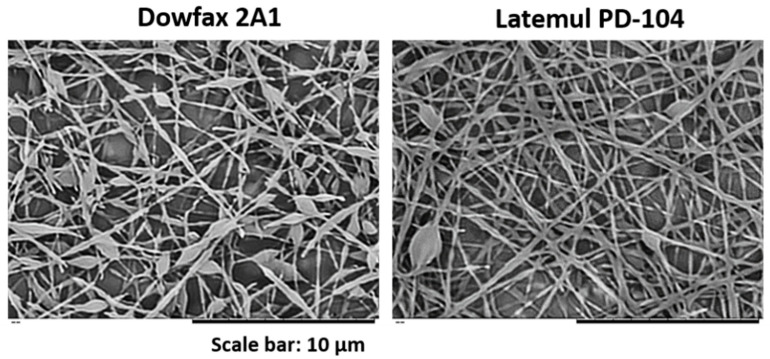
SEM images of fibers obtained from blends of latexes D_2 (Dowfax 2A1 as surfactant) and L_1 (Latemul PD-104 as surfactant) with a PVA/particle ratio of 29/71 wt.%/wt.%. The solids content was 17 wt.% in both cases. The experiment was performed at 20 °C and 55 ± 1 of % R.H.

The effect of the surfactant type used to stabilize the polymer particles was also analyzed. To this end, latexes D_2 and L_1 were compared. These latexes had similar average particle diameter and exactly the same composition. The only difference between them was the surfactant used to stabilize the polymer particles. Latex D_2 was stabilized using 1 wbm % of Dowfax 2A1 and latex L_1 using 2 wbm % of Latemul PD-104 (Figure 9).

Figure 9 shows that more uniform fibers were obtained when Latemul PD-104 was used as surfactant. The electrospinning dispersion containing latexes L_1 and D_2 had similar surface tension values; however, higher conductivity and viscosity values were obtained for the dispersions containing latex L_1 (see Table 5). Thus, the reason for the better-quality fibers obtained when latex L_1 was used was attributed to the higher conductivity and viscosity of the electrospinning dispersions. In fact, the viscosity for the blend containing latex L_1 was above the critical window observed in Figure 3, whereas the one for the blend containing D_2 was below it.

In summary, the changes in the surface chemistry of the polymer particles (by the presence of acid groups or by the used surfactant) can alter the viscosity and the conductivity of the complex electrospinning dispersion and, therefore, strongly influence the final fiber morphology. This is the first time that the effect of the polymer particle surface chemistry on the electrospinning process has been studied.

## 4. Conclusions

Green electrospinning is a relatively new promising technology that consists in the use of an aqueous polymer dispersion (latex) as electrospinning medium with the help of a template polymer. This method is a green, clean and safe technology that allows spinning of hydrophobic polymers using water as an electrospinning medium. In this work, a systematic study that investigates the influence of the template polymer molar mass, the particle/template ratio and the total solids content of the dispersion has been carried out. A critical viscosity window (between 0.14 and 0.23 Pa·s) has been defined for bead formation. Dispersions with viscosity values above this critical window form uniform fibers, whereas the ones with lower viscosities lead to bead formation. Furthermore, the effect of the surfactant used to stabilize the polymer particles, the surface functionality of the polymer particles and the use of a bimodal particle size distribution have been studied for the first time. It has been demonstrated that the viscosity of the initial complex dispersion is affected by the particle size distribution and the surface chemistry of the polymer particles (defined by the used surfactant of the presence of functional groups), and therefore, they have a strong influence on the final fiber morphology. As a conclusion, when working with green electrospinning, all the parameters investigated in this work must be carefully taken into account in order to fine-tune the final fiber morphology. 

## Figures and Tables

**Table 1:** Properties of the commercial polyvinyl alcohol (PVA) used as template polymer, including molecular weight (M_w_) and hydrolysis degree.

Name	Commercial Name	M_w_ (kDa)	Hydrolysis Degree (%)
PVA1	Mowiol 13–88	69.5	88
PVA2	Mowiol 25–88	92.6	88
PVA3	Mowiol 47–88	138.9	88

**Table 2:** Properties latexes in terms of composition, surfactant type and particle diameter (d_p_).

Name	Copolymer	Surfactant	d_p_ (nm)
D_1	MMA/BA	Dowfax 2A1 (1 wbm%)	107 ± 1
D_2	MMA/BA	Dowfax 2A1 (1 wbm%)	192 ± 1
D_3	MMA/BA	Dowfax 2A1 (1 wbm%)	317 ± 4
L_1	MMA/BA	Latemul PD-104 (2 wbm%)	171 ± 1
AA_1	MMA/BA/AA	Dowfax 2A1 (1 wbm%)	139 ± 3

wbm %: weight based monomer %. Dowfax 2A1 was purchased from Dow and Latemul PD-104 from Kao Corporation, Tokyo, Japan.

**Table 3:** Composition of the electrospinning dispersions.

PVA/Particles (wt.%/wt.%) *	Latex (g)/(wt.%)	PVA Solution (g)/(wt.%)	Added Water (g/(wt.%)	Final Electrospinning Dispersion (g)/(wt.%)
50/50	2/16.7	10/83.3	-	12/100
38/62	2/20.8	6/62.5	1.6/16.7	9.6/100
29/71	2/23.8	4/47.6	2.4/28.6	8.4/100
17/83	2/27.8	2/27.8	3.2/44.4	72/100

* wt.%/wt.% refers to the dry PVA/particle ratio.

**Table 4:** Properties the electrospinning dispersions prepared blending latex D_2 with PVA2 at different ratios.

PVA/Polymer Particle (wt.%/wt.%)	Surface Tension (mN/m)	Conductivity (µS/cm)
50/50	44.1 ± 0.03	850
38/62	44.5 ± 0.1	739
29/71	42.6 ± 0.1	569
17/83	46.8 ± 0.1	552

**Table 5:** Properties the electrospinning dispersions prepared blending latexes D_1, AA_1 and L_1 with PVA2 latexes with PVA2 in a PVA/particle ratio of 29/71 wt.%/wt.%. The solids content was 17 wt.% in all the cases.

Latex	Surface Tension (mN/m)	Conductivity (µS/cm)	Viscosity (Pa s)	pH
D_1	49.5 ± 0.1	789	0.19	4.7
AA_1	44.0 ± 0.1	1395	0.35	3.9
L_1	45.0 ± 0.1	1231	0.25	4.2

## Data Availability

The data is available on reasonable request from the corresponding author.

## Supplementary Materials

The following are available online at https://www.mdpi.com/2079-4991/11/3/706/s1, Table S1: Formulation used for the production of the seed, Table S2. Formulation for the second step of the synthesis of latex D_1. Table S3: Formulation for the second step of the synthesis of latex D_2, Table S4: Formulation for the second step of the synthesis of latex D_3. Table S5: Formulation for the synthesis of latex AA_1, Figure S1: Viscosity of electrospinning dispersions prepared blending latex D_2 with PVAs of different molar mass, Figure S2: Viscosity of electrospinning dispersions with different s.c. prepared blending latex D_2 with PVA2 in a PVA/particle ratio of 38/62 wt.%/wt.%, Figure S3: Viscosity of electrospinning dispersions prepared blending latexes D_1, D_2 and D_3 with PVA2 in a PVA/particle ratio of 38/62 wt.%/wt.%, Figure S4: Viscosity of electrospinning dispersions prepared blending latexes D_1, D_3 and a blend of D_1 and D_3 (in a 50/50 wt.%/wt.%) with PVA2 in a PVA/particle ratio of 29/71 wt.%/wt.%, Figure S5: Viscosity of electrospinning dispersions prepared blending latexes D_1, AA_1 and L_1 with PVA2 in a PVA/particle ratio of 29/71 wt.%/wt.%, Figure S6: Particle size distribution of the different latex latexes measured by DLS.
