# Supplementary material for: Green Electrospinning of Polymer Latexes: A Systematic Study of the Effect of Latex Properties on Fiber Morphology
Source: Nanomaterials (Basel). 2021 Mar 11;11(3):706. doi: 10.3390/nano11030706 (PMC7999345; doi:10.3390/nano11030706)
Supplement: Supplementary file 1 [file nanomaterials-11-00706-s001.pdf]

Supplementary Material

Green Electrospinning of Polymer Latexes: A Systematic Study of the Effect of Latex Properties on Fiber Morphology

Edurne Gonzalez *, Aitor Barquero, Belén Muñoz-Sanchez, María Paulis and Jose Ramon Leiza

POLYMAT, Kimika Aplikatua Saila, Kimika Fakultatea, University of the Basque Country UPV/EHU, Joxe Mari Korta Zentroa, Tolosa Hiribidea 72, 20018 Donostia-San Sebastián, Spain; aitor.barquero@ehu.eus (A.B.); belen.munozsa@gmail.com (B.M.-S.); maria.paulis@ehu.eus (M.P.); jrlei-za@ehu.eus (J.R.L.)

* Correspondence: K.Moeller@lmu.de (K.M.); bein@lmu.de (T.B.); Fax: (+49) 89-2180-77622 (T.B.)

1. Synthesis of the Polymer Dispersions.

1.1. Latexes D_1, D_2 and D_3.

The polymer dispersions were synthetized in two steps by seeded semibatch emulsion polymerization in a 250 mL glass jacketed reactor, equipped with reflux condenser, nitrogen inlet and sampling device. The temperature of the reactor was controlled with a thermostatic water bath.

In the first step, the seed latex was synthetized by semibatch polymerization, using the formulation given in Table S1. About 5 mL of water were used to dissolve the initiator (KPS), and the rest of the water and the surfactant (Dowfax 2A1) were loaded to the reactor and the temperature was increased to 70 °C. When the reaction temperature was achieved, the KPS aqueous solution was injected, and after waiting for 10 min the monomer mixture (MMA and BA) was fed using a syringe pump for 120 min at 0.33 mL·min^{-1}. After the feeding ended, it was left 1 h at 70 °C to consume all the monomer. A seed of 20 wt% s.c. and average particle diameter of 60 nm was produced. This seed was used for the synthesis of latexes D_1, D_2 and D_3.

Table S1. Formulation used for the production of the seed.

Chemical	Amount (g)
MMA	20.00
BA	20.00
Dowfax	1.78
Water	160.00
KPS	0.12

In the second step the particles were grown in a semibatch process. The temperature of the reactor and the flow rate of the feeding pumps were controlled by means of an automatic setup that uses the commercial software Camile TG. All the initial charge (the necessary amount of seed and a fraction of the water) was loaded into the rector. When the reaction temperature was achieved (70 °C), a water solution of the initiator was added as a shot. After 10 minutes, the feeding of the rest of the ingredients started.

For the synthesis of latex D_1 only pure monomers were fed using a syringe pump at 0.31 mL·min^{-1} for 120 min. For the synthesis of latexes D_2 and D_3 a preemulsion that consisted of a mixture of the monomers, water and surfactant, was fed, at 1.12 g·min^{-1} for 120 min and 0.8 g·min^{-1} for 240 min, respectively. The formulation for each latex is given in Tables S2-S4.

Table S2. Formulation for the second step of the synthesis of latex D_1.

Reaction steps	Chemical	Amount (g)
Initial charge	seed	60.60
Initiator solution	KPS	0.13
	Water	5.00
Feeding	MMA	17.00
	BA	17.00

Table S3. Formulation for the second step of the synthesis of latex D_2.

Reaction steps	Chemical	Amount (g)
Initial charge	seed	11.60
	Water	50.00
Initiator solution	KPS	0.13
	Water	5.00
Feeding	MMA	48.90
	BA	48.90
	Water	0.98
	Dowfax 2A1	35.80

Table S4. Formulation for the second step of the synthesis of latex D_3.

Reaction step	Chemical	Amount (g)
Initial charge	seed	5.10
	Water	100.00
Initiator solution	KPS	0.13
	Water	5.00
Feeding	MMA	73.80
	BA	73.80
	Water	1.63
	Dowfax 2A1	42.30

1.2. Latex AA_1

Latex AA_1 was also synthetized in a two-step process. In the first step, the seed polymer was produced by miniemulsion polymerization, and, in the second step, the particles were grown in a semibatch process. The same equipment as for the production of latexes in series D was used.

The miniemulsion was obtained by sonicating the mixture of the water (water and Dowfax) and organic (MMA, BA, AA and SA) phases in a Branson Digital Sonifier for 15 min at 70% amplitude and a 0.8 s on and 0.2 s off cycle in an ice bath under magnetic stirring. After obtaining the miniemulsion, it was charged to the reactor and heated to 75 °C before the initiator solution (I) was added as a shot. After 30 min of polymerization, initiator solution (II) was added as a shot and the feeding started (at 0.34 $g{\cdot}min^{-1}$ for 60 minutes). The formulation is given in Table S5.

Table S5. Formulation for the synthesis of latex AA_1.

Reaction step		Chemical	Amount (g)
Initial charge (miniemulsion)	Oil phase	MMA	39.6
		BA	39.6
		AA	0.8
		SA	3.3
	Water phase	Dowfax 2A1	3.6
		Water	110
Initiator solution (I)		Water	5.0
		KPS	0.4
Initiator solution (II)		Water	5.0
		KPS	0.2
Feeding		MMA	19.8
		BA	19.8
		AA	0.4

1.3. Latex L_1

Latex L_1 was synthesized using the same procedure as Aguirreurreta et al. [1]

2. Rheology of Electrospinning Samples.

Figures S1 to S4 present the viscosity results for the different electrospinning dispersions used in this work.

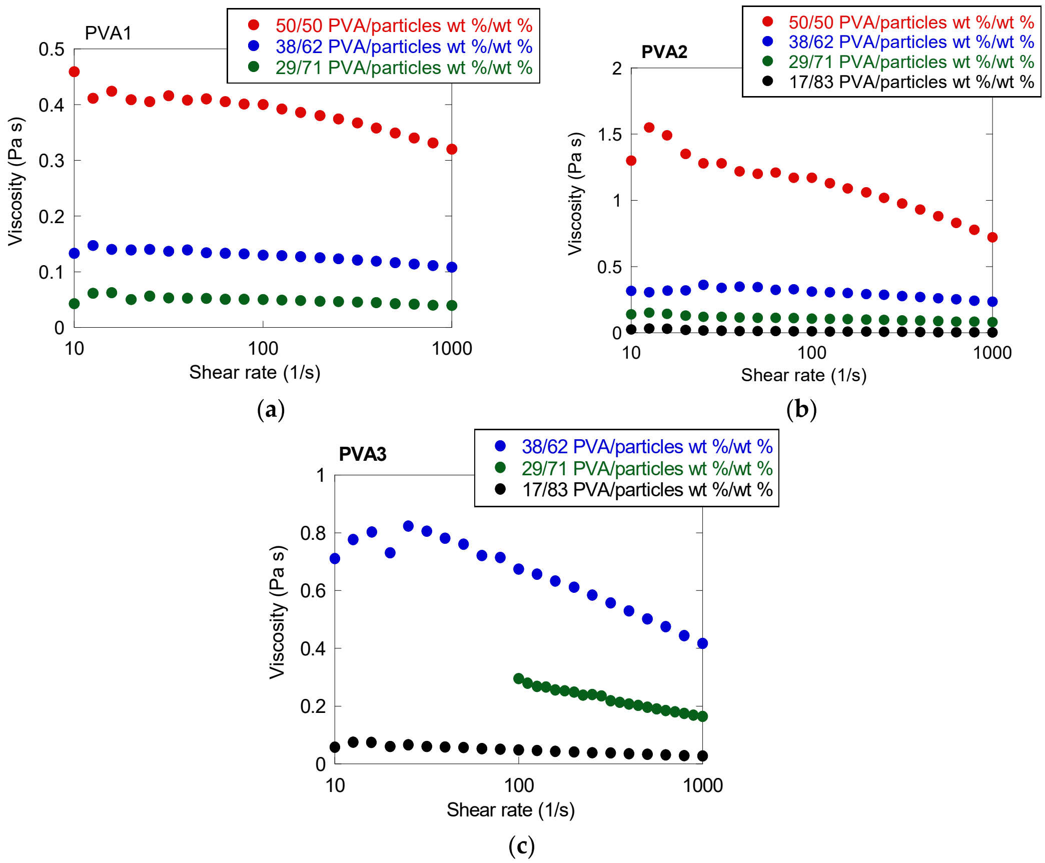

Figure S1. Viscosity of electrospinning dispersions prepared blending latex D_2 with PVAs of different molar mass. (**a**) PVA1, (**b**) PVA2 and (**c**) PVA3.

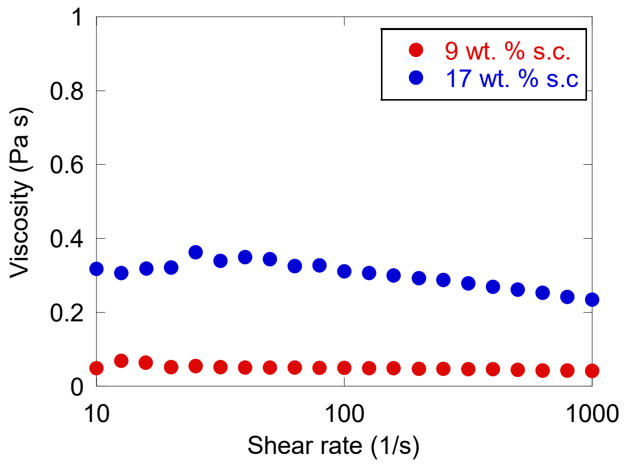

Figure S2. Viscosity of electrospinning dispersions with different s.c. prepared blending latex D_2 with PVA2 in a PVA/particle ratio of 38/62 wt. %/wt. %.

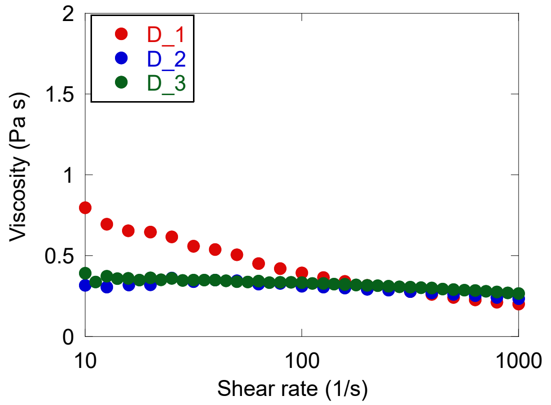

Figure S3. Viscosity of electrospinning dispersions prepared blending latexes D_1, D_2 and D_3 with PVA2 in a PVA/particle ratio of 38/62 wt. %/wt. %.

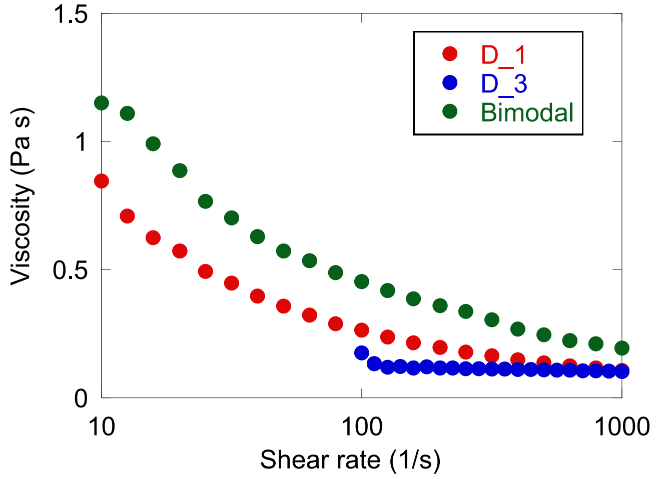

Figure S4. Viscosity of electrospinning dispersions prepared blending latexes D_1, D_3 and a blend of D_1 and D_3 (in a 50/50 wt%) with PVA2 in a PVA/particle ratio of 29/71 wt. %/wt. %.

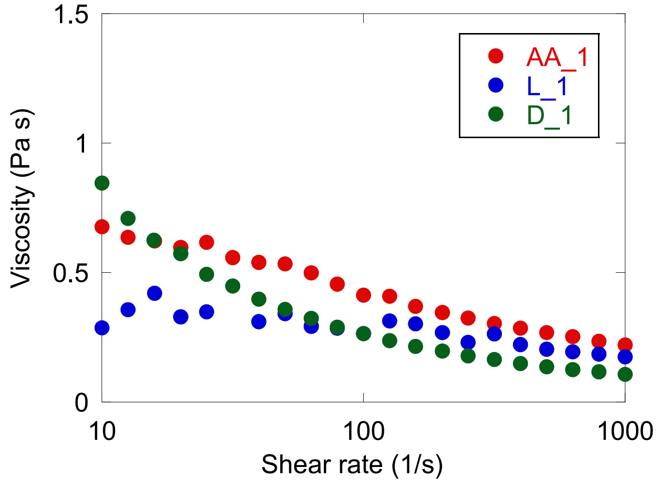

Figure S5. Viscosity of electrospinning dispersions prepared blending latexes D_1, AA_1 and L_1 with PVA2 in a PVA/particle ratio of 29/71 wt. %/wt. %.

Figure S6 shows the particle size distribution (measured by DLS) of the different latexes used in this work.

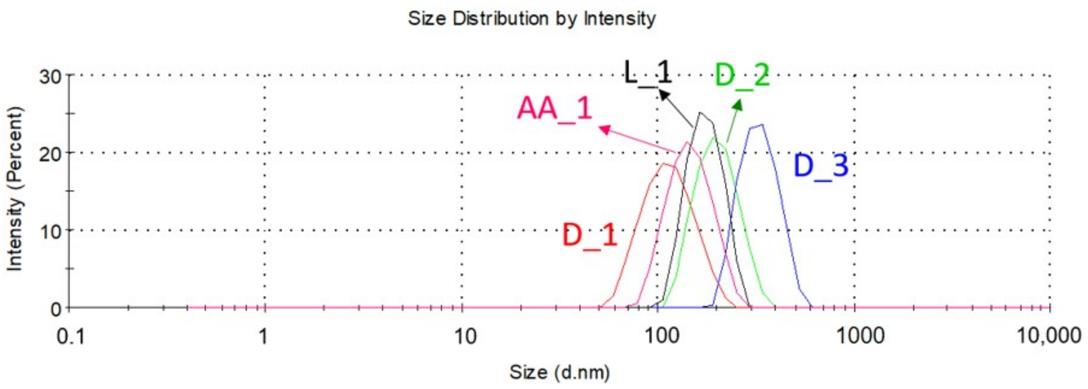

Figure S6. Particle size distribution of the different latex latexes measured by DLS.

References

1. Aguirreurreta, Z.; de la Cal, J.C.; Leiza, J.R. Preparation of high solids content waterborne acrylic coatings using polymerizable surfactants to improve water sensitivity. *Prog. Org. Coatings* **2017**, *112*, 200–209, doi:10.1016/j.porgcoat.2017.06.028.
